# Widespread uncoupling between transcriptome and translatome variations after a stimulus in mammalian cells

**DOI:** 10.1186/1471-2164-13-220

**Published:** 2012-06-06

**Authors:** Toma Tebaldi, Angela Re, Gabriella Viero, Ilaria Pegoretti, Andrea Passerini, Enrico Blanzieri, Alessandro Quattrone

**Affiliations:** 1 Laboratory of Translational Genomics, Centre for Integrative Biology (CIBIO), University of Trento, 38123, Trento, Italy; 2 University of Trento, Department of Information Engineering and Computer Science (DISI), 38123, Trento, Italy; 3 National Research Council, Institute of Biophysics & Bruno Kessler Foundation, 38123, Trento, Italy

**Keywords:** Polysomal, Profiling, Transcriptome, Translational, Control, Translatome

## Abstract

**Background:**

The classical view on eukaryotic gene expression proposes the scheme of a forward flow for which fluctuations in mRNA levels upon a stimulus contribute to determine variations in mRNA availability for translation. Here we address this issue by simultaneously profiling with microarrays the total mRNAs (the transcriptome) and the polysome-associated mRNAs (the translatome) after EGF treatment of human cells, and extending the analysis to other 19 different transcriptome/translatome comparisons in mammalian cells following different stimuli or undergoing cell programs.

**Results:**

Triggering of the EGF pathway results in an early induction of transcriptome and translatome changes, but 90% of the significant variation is limited to the translatome and the degree of concordant changes is less than 5%. The survey of other 19 different transcriptome/translatome comparisons shows that extensive uncoupling is a general rule, in terms of both RNA movements and inferred cell activities, with a strong tendency of translation-related genes to be controlled purely at the translational level. By different statistical approaches, we finally provide evidence of the lack of dependence between changes at the transcriptome and translatome levels.

**Conclusions:**

We propose a model of diffused independency between variation in transcript abundances and variation in their engagement on polysomes, which implies the existence of specific mechanisms to couple these two ways of regulating gene expression.

## Background

In the flow of genetic information, translational control is the level at which reprogramming of cell activities accesses the phenotype, ultimately shaping protein synthesis and therefore, together with the control of protein degradation, quantitative variation of the proteome. Originally studied in early stages of development in oocytes and embryos [1,2], translational control has been increasingly recognized as a very general feature of eukaryotic cells, extensively present also in mature tissues. This process is orchestrated by incoming cell stimuli which elicit largely unknown transduction pathways, affecting primarily translation initiation, i.e. the loading of ribosomes on messenger ribonucleoprotein particles (mRNP) to form polysomes, and secondarily translation elongation [3,4]. The ways in which these stimuli influence polysome formation involve “general” translation factors as eIF4E, eIF4G, eIF4A and PABP, allowing mRNA circularization and ribosome scanning, and more specialized factors acting on sequences found primarily in the 5’ or 3’ untranslated regions (UTRs) of mRNAs. These latter factors belong to the two classes of RNA binding proteins (RBPs) and noncoding RNAs (ncRNAs), among which microRNAs (miRNAs) are an intensively studied subclass. In the human genome the predicted genes coding for proteins involved in translational control are around a thousand and the number of miRNAs, proven to be able to modulate translation [5,6], is estimated between one and two thousands [7]. Furthermore, by recent transcriptome high-sensitivity sequencing scannings, the human ncRNA collection has risen to comprise around five thousands ncRNAs [8], to which the 18,000 [9] processed pseudogenes have to be added because they also can interfere with gene expression [10]. If even a small fraction of these ncRNAs was involved in modulating translation, the amount of macromolecules potentially able to operate at the interface between mRNA and proteins would be extremely high. Moreover, recent findings reveal the presence in eukaryotic cells of cytoplasmic RNA-containing granules (processing bodies, stress granules and other types) composed of aggregates of mRNPs where mRNA decay, editing and storage can take place [11-13]. These granules can generate a bidirectional flow of mRNAs with polysomes [14-16].

Given this complex layer of activities in the cytoplasm, we set the goal to estimate the relationship between fluctuations of mRNA levels in the cell and fluctuations of the fraction of mRNAs available for translation after a stimulus, which to our knowledge has never been addressed with a population-based approach. The degree of change in translation-engaged mRNAs can be estimated by extracting mRNAs organized in polysomes by a classical separation technique, velocity sedimentation by sucrose gradients, and profiling them in parallel with total mRNA [17].

By measuring the total mRNAs of cells (the transcriptome) and the polysomally-loaded mRNAs (the translatome) after a growth stimulus, we obtained a picture of overall mismatching between the two changes for the majority of genes, to which we refer as “uncoupling” in the mRNA behavior. This was confirmed studying a number of other available profiles coming from very diverse experiments and kinetics. The marked, general uncoupling between transcriptome and translatome gene expression changes allowed us to propose a biological model by which the machineries responsible for mRNA availability in the cytoplasm and for mRNA engagement in translation lack overall dependency, therefore questioning the notion of continuity in the control of the flow of gene expression.

## Results

### Profound uncoupling between transcriptome and translatome gene expression variations upon EGF stimulation of HeLa cells

**Figure 1:**
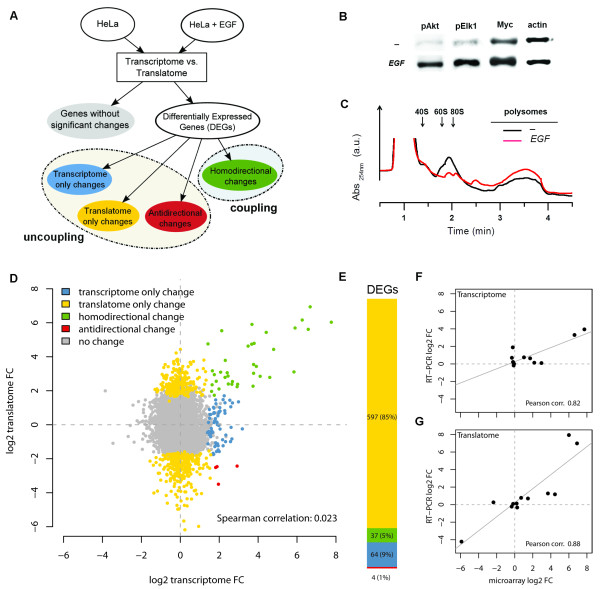
**EGF treatment of HeLa cells induces extensive uncoupling between transcriptome and translatome gene expression variations.** (**A**) Flowchart of differential expression analysis between transcriptome and translatome after EGF treatment and definition of uncoupling. Uncoupling qualifies genes classified as DEGs (differentially expressed genes) with significant variations only in the transcriptome (in cyan), only in the translatome (in yellow) and with opposite significant variations between transcriptome and translatome (in red). Coupling qualifies genes classified as differentially expressed (DEGs) by both transcriptome and translatome profile comparisons and with homodirectional changes (in green). (**B**) Western blots indicating the activation of the EGFR signaling pathway by the increase of known EGFR mediators and targets: phosphorylated Akt1, phosphorylated Elk1 and Myc. (**C**) Comparison between sucrose gradient profiles of HeLa cells without EGF (in black) and with EGF (in red). (**D**) Scatterplot of transcriptome and translatome log2 transformed fold changes, showing genes belonging to the coupling and uncoupling categories as defined in panel A. Spearman correlation between fold changes is also shown. (**E**) Barplot highlighting the uncoupling value between translatome and transcriptome DEGs. The number of DEGs and the corresponding percentages are displayed following the same colour scheme adopted in the rest of the figure (**F-G**) Scatterplot showing correlation between transcriptome (**F**) and translatome (**G**) log2 transformed fold changes derived from microarray hybridizations and quantitative RT-PCR on a set of twelve genes, displayed as black dots. Regression lines are drawn in grey.

To address the impact of translational regulation in reshaping transcriptome profiles we chose a classical paradigm of cellular reprogramming of gene expression, Epidermal Growth Factor (EGF) treatment of starved cells. This stimulus elicits a well-known chain of intracellular transduction events, resulting in a complex phenotypic spectrum of changes with prevalent induction of cell growth and proliferation [18,19]. As outlined in Figure 1A, we treated HeLa cells under serum starvation with EGF for 40 minutes (final concentration of 1 μg/ml). The activation of the EGF signalling cascade is proved by an increased phosphorylation of AKT and ELK1, known EGFR downstream effectors [20,21], and by an increase of MYC, an early EGF transcriptional target [22] (Figure 1B). Consistently with an overall engagement of the translational machinery by EGF, the absorbance profiles obtained after sucrose gradient centrifugation of lysates from EGF-treated compared to control cells show a clear increase of RNA associated to the polysomal fractions and a concomitant reduction of RNA present in the subpolysomal portion of the gradient (Figure 1C). We then profiled by gene expression arrays both the transcriptome and the translatome, before and after 40 minutes of EGF treatment. Microarray results were validated with quantitative real time PCR on a selected subset of twelve genes, showing a good concordance between the two independent sets of measurements (Figure 1F-G, in Additional file 1: Table S1): Pearson correlation was 0.82 for transcriptome data and 0.88 for translatome data. Differentially expressed genes (DEGs) upon EGF treatment were detected from microarray data with the RankProd algorithm [23] separately at the transcriptome and translatome level. This allowed us to obtain a simple classification of DEGs into “coupled” or “uncoupled”, based on the concordance of their variation between the transcriptome and the translatome (Figure 1A). We consider the DEGs coupled if they show a significant change in both the transcriptome and the translatome and if the change is homodirectional (always displayed in green in Figure 1A, 1D and 1E). They are instead scored as uncoupled if (a) they change significantly in both the transcriptome and the translatome but in an antidirectional way (always displayed in red throughout the paper), (b) they change significantly only in the transcriptome (always displayed in cyan) and (c) they change significantly only in the translatome (always displayed in yellow). Following these criteria, the proportion of coupled DEGs observed in our experiment is only 4.8% (37 genes), against the overwhelming 95.2% proportion of uncoupled DEGs (665 genes; Figure 1E, Additional file 2). Furthermore, among the uncoupled DEGs, purely translatome DEGs are nine times more frequent than purely transcriptome DEGs (597 against 64) and transcriptome DEGs result to be exclusively upregulated. Plotting translatome versus transcriptome fold changes makes clear that the variations in mRNA abundance are poorly correlated with the variations in mRNA polysomal engagement (Figure 1D). Therefore, treatment of HeLa cells with a well-known growth factor results to target mostly translation, with a negligible concordance between the two levels of regulation. We next sought to determine if the observed differences between the two profiles were also reflected in variations of predicted cellular processes and activities. DEGs were annotated by sequence, protein domain, phylogenetic and functional descriptors: PIR resource [24], InterPro database [25], COG database [26], KEGG [27] and Biocarta pathway databases, Gene Ontology [28]. The high degree of uncoupling was confirmed by enrichment analysis of the transcriptome and translatome DEGs, resulting in sharply distinct patterns of significant terms, with only 27 common terms (17%), 90 transcriptome-specific terms and 43 translatome-specific terms ( Additional file 1: Figure S1 and Additional file 3).

### The high degree of uncoupling between transcriptome and translatome variation profiles is a general feature of the control of gene expression in mammalian cells

**Figure 2:**
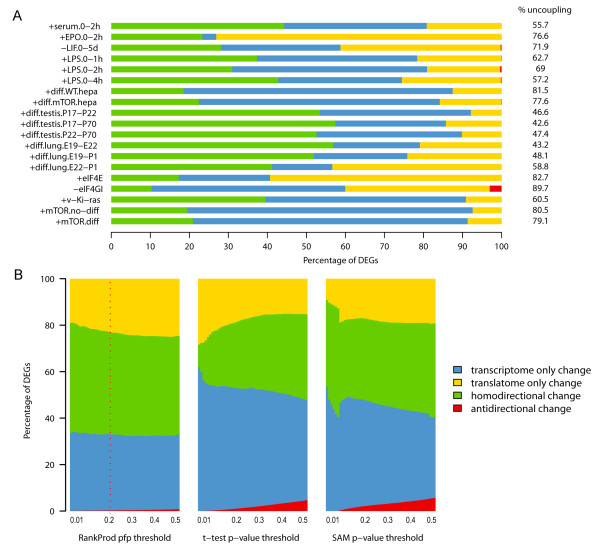
**Widespread gene expression uncoupling is a general and recurring phenomenon in all transcriptome-translatome profiling datasets. (A)** Barplot displaying the degree of uncoupling between transcriptome and translatome DEGs for each dataset. Collected datasets are labelled by short names as explained in Table 1. Bar lengths show the relative proportion of DEGs in the four classes defined in Table 1. The corresponding percentages of uncoupled DEGs are shown on the right. **(B)** Uncoupling estimate is independent from the significance threshold and the algorithm used for calling DEGs. Percentage of DEGs detected by the comparison (homodirectional change in green, antidirectional change in red) between both transcriptome and translatome profiles, DEGs detected by the transcriptome comparison only (in cyan) and DEGs detected by the translatome comparison only (in yellow) were computed over all the datasets described in Table 1. Three algorithms are shown: RankProd, *t*-test and SAM. Inside each barplot the significance thresholds ranges from 0.01 to 0.5. In the barplot generated with RankProd the red vertical dashed line indicates the 0.2 significance threshold used to detect DEGs throughout the analysis. For *t*-test and SAM a Benjamini-Hochberg multiple test correction was applied to the resulting p-values.

To test whether our observation of strong discordance between the variations of total mRNAs and polysome-associated mRNAs could be of some generality in mammals, we systematically reanalyzed already published experiments in which both the transcriptome and the translatome (the last always isolated by sucrose gradient) were profiled in mammalian cells and tissues. We selected the experiments according to stringent quality standards (see Methods) to ensure technical comparability between different studies. Among an initial database of 16 mammalian studies, we finally identified 10 experiments involving observation of different treatments and processes in human, mouse and rat cells and tissues, giving a total of 19 paired transcriptome/translatome datasets. The profiles belonged to three types of experiments: short-term treatments with extracellular stimuli (4 experiments, 6 paired datasets), differentiation processes in cells and tissues (3 experiments, 8 paired datasets) and induced genetic alterations of the translational machinery (4 experiments, 5 paired datasets). The experiments are briefly described in Table 1 and extensively annotated in Additional file 4. All the microarrays used in the experiments belong to the Affymetrix platform: this decreases the risk of introducing in the following analyses cross-platform biases due to different manufacturing technologies ( Additional file 1: Table S2 and Figure S3). Raw microarray data were subjected to the same normalization and DEGs selection procedure previously described for the EGF experiment (processed data in Additional file 5). To measure the significance of differential expression, we chose the RankProd algorithm because, transforming the actual expression values into ranks, it offers a way to overcome the heterogeneity among multiple datasets and therefore to extract and integrate information from them [23]. In order to keep a methodological homogeneity, we also chose to apply for all the datasets the same significance threshold. To quantify the transcriptome/translatome uncoupling for each paired dataset, we calculated the percentage of uncoupled DEGs, which outnumbered coupled DEGs in two thirds of the analyzed datasets (14 out of 19 comparisons, Figure 2A) the percentage of uncoupled DEGs ranging from 43.2% to 89.7% with an average of 64.8%. Conversely, the percentage of coupled DEGs ranges from a minimum of 10.3% to a maximum of 57.4%, with an average of 35.2%. Importantly, these relative proportions between uncoupled and coupled DEGs are stable even when using different significance thresholds to identify DEGs, or alternative DEG detection methods (Figure 2B and in Additional file 1: Figure S2). As alternatives we used *t*-test and SAM [29], by which we can show an even more extensive uncoupling than by RankProd. Therefore, this broad analysis confirmed that the marked uncoupling between transcriptome and translatome profiles is a feature far from being confined to short-time treatment of HeLa cells with EGF, assuming instead the dimension of a general principle describing change of gene expression in mammals*.*

**Table 1:** Description of the datasets used for the analysis

**Short name**^**a**^	**Description**	**Biological source**	**Reference**	**Data ID**^**b**^	**Chip**^**c**^	**Cluster**
**+serum.0-2 h**	serum starvation release	*Mus musculus*	PMID: 17405863	GSE7363	MG_U74Av2	extracellular signalling
**+EPO.0-2 h**	erythroid EPO deprivation release	*Mus musculus*	PMID: 18625885	E-MEXP-1689	MG_U74Av2	
**-LIF.0-5d**	stem cell differentiation through LIF removal	*Mus musculus*	PMID: 18462695	GSE9563	Mouse430_2	
**+LPS.0-1 h**	macrophage LPS treatment (1 h)	*Mus musculus*	PMID: 18230670	GSE4288	Mouse430_2	
**+LPS.0-2 h**	macrophage LPS treatment (2 h)	*Mus musculus*	PMID: 18230670	GSE4288	Mouse430_2	
**+LPS.0-4 h**	macrophage LPS treatment (4 h)	*Mus musculus*	PMID: 18230670	GSE4288	Mouse430_2	
**+diff.WT.hepa**	differentiation of WT hepatocytes	*Homo sapiens*	PMID: 18221535	E-MEXP-958	HG-U133A	differentiation	
**+diff.mTOR.hepa**	differentiation of mTOR activated hepatocytes	*Homo sapiens*	PMID: 17483347	E-MEXP-958	HG-U133A		
**+diff.testis.P17-P22**	testis differentiation (5d)	*Mus musculus*	PMID: 16682651	GSE4711	MOE430A		
**+diff.testis.P17-P70**	testis differentiation (53d)	*Mus musculus*	PMID: 16682651	GSE4711	MOE430A		
**+diff.testis.P22-P70**	testis differentiation (48d)	*Mus musculus*	PMID: 16682651	GSE4711	MOE430A		
**+diff.lung.E19-E22**	lung differentiation (3d)	*Rattus norvegicus*	PMID: 18952566	GSE12153	Rat230_2		
**+diff.lung.E19-P1**	lung differentiation (embrionic vs postnatal)	*Rattus norvegicus*	PMID: 18952566	GSE12153	Rat230_2		
**+diff.lung.E22-P1**	lung differentiation (embrionic vs postnatal)	*Rattus norvegicus*	PMID: 18952566	GSE12153	Rat230_2		
**+eIF4E**	eIF4E overexpression	*Homo sapiens*	PMID: 17638893	GSE6043	HG-U133_Plus_2	translational machinery alteration	
**-eIF4GI**	eIF4GI depletion	*Homo sapiens*	PMID: 18426977	GSE11011	HG-U133A_2		
**+v-Ki-ras**	v-Ki-ras transformation	*Homo sapiens*	PMID: 16446406	E-MEXP-461	HG_U95Av2		
**+mTOR.no-diff**	mTOR activation of proliferative hepatocytes	*Homo sapiens*	PMID: 17483347	E-MEXP-958	HG-U133A		
**+mTOR.diff**	mTOR activation of differentiated hepatocytes	*Homo sapiens*	PMID: 17483347	E-MEXP-958	HG-U133A		

(a) short name specifying exposure to or subtraction from (+ or –) a broadly defined perturbation agent, perturbation agent name, experimental time.

(b) dataset reference on GEO or ArrayExpress.

(c) all the chips belong to the Affymetrix platform.

### Ontological enrichment and pathway analysis of transcriptome and translatome variations predict very different phenotypes

**Figure 3:**
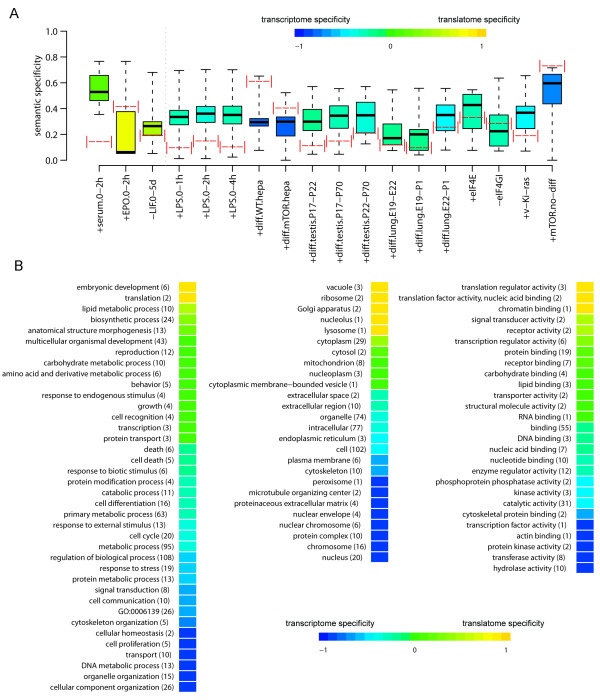
**Uncoupling between transcriptome and translatome is conserved in the enriched biological themes**.** (A)** Summary of semantic specificity estimates (based on the optimized quantification of semantic specificity described in SI Materials and Methods). Red dotted lines represent semantic specificity estimates relative to the transcriptome and translatome comparisons within all datasets. Box and whisker plots show the reference distributions of semantic specificities (whiskers indicating minimal and maximal distribution values), characteristic of each dataset and reflecting semantic specificity estimates between the transcriptomes of unrelated dataset pairs. A semantic specificity falling within or below the reference distribution is indicative of very poor semantic similarity between the transcriptome and the translatome in a dataset pair. The color associated to the box of each dataset pair corresponds to the normalized difference between the number of GO terms over-represented only at the translatome level and the number of GO terms over-represented only at the transcriptome level, a quantity ranging from −1 (all the terms are enriched only at the transcriptome level, in blue) to 1 (all the terms are enriched only at the translatome level, in yellow). This measure is positive for the first three datasets on the left and negative for all the others (divided by a vertical dashed line in the figure). Having no overrepresented ontological terms, the dataset + mTOR.diff is not displayed. **(B)** For each GO term the transcriptome and translatome specificity degrees are calculated as the ratio between the number of datasets in which the term is transcriptome or translatome specific and the number of datasets in which the term is overrepresented. Terms are grouped into the broader GOslim categories and the median specificity values are calculated. The number of GO terms grouped in each GOslim category is specified in round brackets. Within each of the three GO domains (from left to right: Biological Process, Cellular Component and Molecular Function), categories are sorted from the most translatome-specific (in yellow) to the most transcriptome-specific (in blue).

We were then interested in estimating the impact of gene expression uncoupling on the cell activities ascribed to the transcriptome and the translatome DEGs, when studying the whole collection of experiments. All the lists of DEGs from the dataset pairs were independently subjected to ontological enrichment analysis as for our EGF experiment (data available in Additional file 6). We tested whether the gene expression uncoupling between transcriptome and translatome can originate a semantic specificity between the two relative sets of enriched ontological terms. Two measures of semantic specificity were adopted. The first measure is based on the simple enumeration of cell activities that, as an effect of uncoupling, resulted enriched uniquely in the transcriptome or in the translatome DEGs (Figure 3A, color code of the boxplot). Transcriptome specificity is higher (87%) than translatome specificity in the large majority of dataset pairs, except for three of them related to short-term cell treatments. The second measure of semantic specificity accounts also for semantic similarity relationships between not identical ontological terms (see Methods), and was applied to all the dataset pairs (red bars in Figure 3A). Semantic specificities were low, with an average value of 0.26 and with 16 dataset pairs falling below the midrange value of 0.5. To further estimate the extent of the distance between the transcriptome and the translatome of each experiment, we compared the semantic specificity measures with a reference distribution, calculated as the set of semantic specificities between the transcriptome of each dataset pair and the transcriptome of all the other datasets. Since the datasets collected were largely heterogeneous, they were assumed to show a low semantic relationship between their transcriptome DEGs. Surprisingly, the semantic specificity observed between the transcriptome and the translatome in all the dataset pairs except one was found within or below the distribution, and in 13 of them below the distribution median (Figure 3A). Taken together, the results show unexpectedly weak semantic similarity between the transcriptome and the translatome ontological enrichments of all the considered experiments.

Finally, we wanted to derive from the global ontological analysis those cell activities more specifically characterizing transcriptome DEGs compared to translatome DEGs and vice versa. To provide a general view, individual over-represented GO terms from all dataset pairs were mapped to GOslim [30], a simplified version of GO. A clear outcome was that half of the translatome-specific terms (including *translationtranslation regulator activitytranslation factor activityribosome*) were exclusively translation-related (Figure 3B). This result provides additional support to the notion of independent transcriptome and translatome controls of gene expression variations.

For each dataset, lists of transcriptome and translatome DEGs were subjected to further annotation with the Ingenuity Pathway Analysis (IPA) library of canonical pathways (data available in Additional file 7). The significance of the association between the DEGs and the canonical pathways was measured with the Fisher’s exact test, and a 0.05 cut-off on the Benjamini-Hochberg corrected p-value was used to identify significantly enriched pathways. Comparing the number of pathways that resulted enriched uniquely in the transcriptome or in the translatome DEGs, we had another proof that the gene expression uncoupling between transcriptome and translatome is extended to a functional specificity between the two relative sets of enriched pathways (Additional file 1: Figure S5). Across all the dataset pairs, 97 pathways (22%) were significantly enriched only in transcriptome DEGs, 139 pathways (31%) only in translatome DEGs and 206 pathways (47%) in both transcriptome and translatome DEGs. In 14 out of the 16 datasets with at least one enriched pathway, the number of specific pathways exceeds the number of common pathways.

The Ingenuity Knowledge Base was employed to build networks from the lists of transcriptome and translatome DEGs for each dataset. Networks were generated using experimentally validated direct interactions among DEGs (data available in Additional file 8). Cellular functions associated to networks, based on the functional annotation of their genes, were ranked according to their translatome specificity ( Additional file 1: Table S3). *RNA post-transcriptional modification,* again an mRNA related theme, resulted as a cellular function mainly associated to translatome networks.

### Transcriptome and translatome variations are globally not dependent

**Figure 4:**
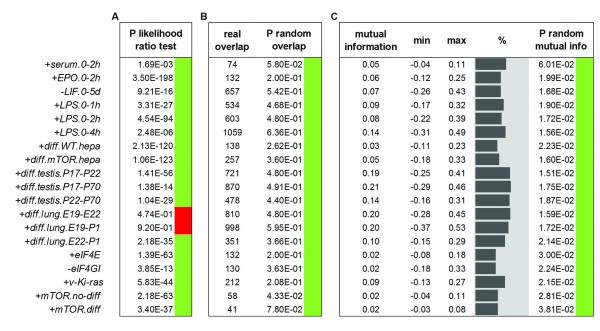
**Gene expression uncoupling is consistent with a hypothesis of lack of dependence between transcriptome and translatome variations.** Results in agreement with the lack of dependence hypothesis are labeled with a green square, while results rejecting the lack of dependence hypothesis are labeled with a red square. **(A)** Likelihood Ratio p-values, testing the hypothesis that the numbers of DEGs in the transcriptome and the translatome are different, result significant for 17 of 19 datasets (P < 0.01). **(B)** The overlap observed between transcriptome and translatome DEGs is never significantly higher than its random estimate (random overlap P > 0.01 in 19 out of 19 datasets). **(C)** Mutual information observed between transcriptome and translatome is never significantly higher than its random estimate (random mutual information P > 0.01 in 19 out of 19 datasets). Theoretical mutual information minima and maxima are also calculated for each dataset as explained in Methods. The positions of the real mutual information values inside the range defined by the theoretical minima and maxima are visualized as grey histograms.

Having shown the high level of uncoupling between transcriptome and translatome variations by either a gene-oriented and a function-oriented perspective, we speculate that these variations could be controlled by largely independent regulatory mechanisms. If confirmed, this hypothesis would falsify the conventional model of gene expression change where transcriptome fluctuations induced by regulated mRNA synthesis or degradation are implicitly considered determinants of translatome changes, through “mass effects” of increased or decreased mRNA quantities on polysomal occupancy [31]. Indeed, the results of three different statistical tests carried out on the available DEG profiles support a counterintuitive model of transcriptome and translatome relative autonomy (Figure 4). The conventional dependency model reasonably generates the following expectations: (1) the total number of translatome DEGs should be dependent on the total number of transcriptome DEGs, (2) significant variations of expression of a gene in the transcriptome should be reflected in the translatome, and therefore transcriptome DEGs should overlap translatome DEGs in a statistically significant manner. Neither expectation was confirmed by our analysis. In fact, the likelihood ratio test clearly rejected the first expectation, by supporting the notion that the numbers of transcriptome and translatome DEGs are independent in 17 out of the 19 comparisons (Figure 4A). Furthermore, when we tested the second expectation, we found the observed overlap between transcriptome and translatome DEGs to be comparable with the overlap deriving from random sampling of gene variations of expression, never passing a 0.01 p-value threshold for significance by standard non-parametric bootstrap (Figure 4B). To further assess this strong indication of independence, we finally estimated the mutual information between transcriptome and translatome variations, modeled as binary variables. Across all comparisons mutual information values ranged from 0.02 to 0.21, with an average value of 0.09. When we took into account the minimal and maximal mutual information values allowed by the frequencies of DEGs in each dataset pair (corresponding respectively to the event of null overlap and complete overlap between transcriptome and translatome DEGs), the observed mutual information values were not found to deviate from the overall midrange values (mean absolute deviation 0.08). The lack of substantial mutual dependence between transcriptome and translatome DEGs was confirmed by the fact that the observed mutual information values never significantly exceed the corresponding values in random bootstrapping samples (0.01 significance threshold; Figure 4C).

Past studies employing yeast and reticulocyte lysates [32-35] have claimed that mRNAs have to numerically compete to gain access to ribosomes and to form polysomes. According to this view, polysomes should generally buffer transcriptome variations, except for those transcripts associated to trans-acting factors specifically increasing their probability of access to polysomes. To verify this hypothesis in our mammalian datasets, we counted the number of translationally enhanced (polysomal mRNA fold change > total mRNA fold change) and translationally buffered (polysomal mRNA fold change < total mRNA fold change) mRNAs across all the 19 dataset pairs. Since the proportions are roughly 50% and 50% (46% with decreased polysomal access, 54% with increased polysomal access) without a significant majority of genes buffered at the polysomal level, we suggest that in mammalian cells the competition of mRNA for ribosomes is not a general driving force regulating translation, unless the action of trans factors promoting polysome formation has the same magnitude of the polysomal competition effect.

In conclusion, we suggest that by analyzing the available data with different approaches, the mRNA production/degradation and the mRNA access to translation appear to be globally regulated not in an interdependent way.

## Discussion

The conceptualization which framed molecular genetics studies for four decades is the so-called central dogma [36], representing the forward flow of gene expression from DNA to mRNA to proteins through transcription and translation. This directional flow can easily be viewed as an assembly line in which the translation step is automatically determined by the availability of mRNAs produced by the transcription step. Following this scheme, changes in the quantities of an mRNA species due to changes in its transcription and/or degradation rate after a stimulus determine changes in its translation rate.

Prompted by the recent appreciation of translational control being more widespread than originally thought [37], and by the discovery of universal cytoplasmic foci of mRNA accumulation [38], in this work we wanted to address the quantitative population changes induced by a cell stimulus between the cytoplasmic mRNAs and those mRNAs supposed to be actively engaged in translation because part of polysomes. Inspired by the results of an experiment of EGF treatment on HeLa cells, we extended the analysis to a number of available published data on mammalian systems, all obtaining the translatome measures after sucrose gradient separation of polysomes. Therefore the outcome proposed, deriving from 8 treatments and 2 developmental assays performed on 10 different types of mammalian cells, can be likely regarded as general for mammals.

We found that the degree of uncoupling between the transcriptome and the translatome was higher than the degree of coupling, both in terms of single transcripts undergoing changes in levels and in terms of the ontological enrichment of the corresponding proteins. A striking result of the ontological analysis is that the transcriptome variation profiles of two different, unrelated experiments are as diverse as each of them compared with the corresponding translatome variations of the same experiment (Figure 3A). From this result we derive a message of partiality of transcriptome data in representing cell phenotypes, no matter how much quantitatively accurate. We also observed that a general tendency to establish autoregulatory or crossregulatory loops should be a specific feature of mRNAs and proteins involved in translation. In fact, among the mRNAs that change their abundance purely at the translatome level, we observe a strong enrichment in encoded translation-related proteins. This finding extends previous observations of self-regulating translation activities, such as the well-known example of the TOP genes, that are involved in the translation basal machinery and at the same time are regulated at the translational level after cell growth stimuli [39]. This observation provides a first clue for understanding the independence of translatome changes by the transcriptome: following cell stimuli which act on their expression, many genes coding for components of the translational machinery do not undergo any change in their mRNA levels, but only variations in the rate of polysomal loading of these mRNAs. Moreover, we know that several mammalian RBPs bind their own mRNA and the mRNAs encoding other RBPs, regulating their stability and translation [40]. In agreement, reconstruction in the yeast of RBP expression networks suggests that at least one third of the studied proteins post-transcriptionally auto regulate themselves, acting as network hubs [41]. In metazoan a possible strong evolutionary pressure for the wiring of these RBP-mediated post-transcriptional looping circuits could derive from the need to regulate protein synthesis of maternal mRNAs in oocytes and in early embryo development, a well-studied process in *C. elegans* and *Drosophila*[42]. A stimulating framework to explain the complexity and the relative independence of post-transcriptional networks from transcriptional events is proposed by Keene with the concept of post-transcriptional regulons, clusters of discrete mRNAs co-regulated by the same set of RBPs in order to orchestrate complex cellular functions [43,44].

Indirect observations sustaining the view of divergence between the transcriptome and the translatome come instead from *en-masse* analyses comparing absolute mRNA and protein abundance. While in many prokaryotic and eukaryotic systems mRNA levels can describe no more than 50% of protein levels [45,46], in a recent work [47] on human tumor cells the matching is lower (Pearson correlation 0.29) and translation-related features (as coding sequence, 5’ UTR, 3’ UTR lengths, presence of upstream open reading frames, density of secondary structures in the 5’ UTR, amino-acid composition) contribute with 30% to the predictability of protein concentration. Similar conclusions, with a predominant role in control given to translation, were drawn from a quantitative model based on mRNA and protein abundances, synthesis rates and half-lives in mouse fibroblasts and human breast cancer cells [48]. Protein abundances are also more conserved than total mRNA abundances among different taxa, suggesting that transcriptome networks are less affected by evolutionary pressure than proteome networks [49].

Direct transcriptome to translatome comparison studies after severe stresses in yeast provide a picture of a marked translational shutdown from which highly concordant homodirectional changes emerge for some genes [50-53]. Exposing yeast to mild stresses, instead, produces a response characterized by a high level of uncoupling [53]. These last mild perturbations and their dynamic effects can be better assimilated to the stimuli analyzed here in mammalian cells. A statistical treatment of the populations of mRNAs undergoing variations in our collection of comparable dataset pairs provides the falsification of a model of straight dependency between translatome changes from transcriptome changes. As a possible alternative, a parsimonious model in line with these data could postulate a general orthogonality between the mechanisms controlling transcript levels and translation in mammalian cells. In other words, changes in abundance of a given mRNA do not determine per se any effect on changes in its polysomal engagement. While mRNA abundance is controlled in a sequence-dependent way by its rate of transcription and degradation, polysomal engagement of mRNA is determined by translation factors interacting with sequence and structural motifs present in the mRNA itself [54]. These controls do not depend on mRNA abundance if not following titration of the trans-acting factors involved, as recently shown for miRNAs [55]. The view proposed by this model could speculatively match that of the proposed stochastic, “burst-like” nature of transcription [56], characterized by variable kinetics and refractory periods [57], producing therefore a noisy transcriptome which could be later shaped into a more stable proteome by translational control.

Following this parsimonious model the observed degree of coupling between fluctuations of the mRNA levels in the cells and fluctuations in productive ribosome engagement should be an effect of specific mechanisms of molecular pairing between mRNA steady state determinants (i.e., controls of chromatin remodeling, transcription and mRNA decay) and regulation of translation of the same mRNA. The prediction emerging from this study is that a variety of coupling mechanisms, some of which already described [58,59], should be active in mammalian cells to orchestrate cell and tissue primary programs.

## Conclusions

Our study estimated the genome-wide correlation between changes in mRNA abundance and mRNA polysomal loading in an unprecedentedly large collection of mammalian cells and tissues subjected to heterogeneous stimuli. From our results we conclude that the control of gene expression at the polysomal level is pervasive with no exceptions, and genes whose expression changes homodirectionally at the transcriptome and the translatome level represent a minority of those perturbed by the stimuli. From a statistic point of view the variations in the degree of mRNA polysomal loading are, on the whole, independent from variations in mRNA abundance. This independency is further extended to the cell activities inferred from the ontological analysis of transcriptome and translatome differentially expressed genes, with a clear tendency of translation-related genes to be controlled purely at the translational level without modifications in the levels of their transcripts.

## Methods

### EGF treatment of HeLa cells

HeLa CCL-2 cells were cultured in DMEM supplemented with 10% FBS, 2mM glutamine, 100 units/ml penicillin, and 100 mg/ml streptomycin at 37 °C, 5% CO_2_. Cells were seeded on adherent plates and serum starved for 12h with DMEM, 0.5% FBS, 2mM glutamine. Cells were treated for 40 minutes with recombinant human Epidermal Growth Factor (EGF from RD Systems, Minneapolis) at the final concentration of 1 μg ml^-1^. Cell lysates were collected before (t=0 min) and after (t=40 min) EGF treatment. For the total and polysomal RNA extraction, 3 × 105 cells/well (6 well-plates) and 1.5 × 106 cells/dish (10mm dishes) were seeded, respectively, in order to have the same concentration of cells and the same surface density on the dishes). All experiments were run in biological triplicates.

### Total RNA extraction

Total RNA was extracted using the TRIZOL reagent according to the manufacturer's protocol. RNA was quantified using a spectrophotometer and its quality was checked by agarose gel electrophoresis and by the Agilent 2100 Bioanalyzer platform, following the manifacturer’s guidelines for sample preparation and analysis of data (Agilent 2100 Bioanalyzer 2100 Expert User's Guide).

### Polysomal RNA extraction

Cells were washed once with phosphate buffer saline (PBS + cycloheximide 10 μg ml^-1^) and treated directly on the plate with 300 μl lysis buffer [10 mM NaCl, 10 mM MgCl_2_, 10 mM Tris–HCl, pH 7.5, 1% Triton X-100, 1% sodium deoxycholate, 0.2 U μl^-1^ RNase inhibitor (Fermentas), cycloheximide 10 μg ml^-1^ and 1 mM dithiothreitol] and transferred to an Eppendorf tube. After a few minute incubation on ice with occasional vortexing, the extracts were centrifuged for 5 min at 12,000 g at 4 °C. The supernatant was stored at −80 °C or loaded directly onto a 15–50% linear sucrose gradient containing 30 mM Tris–HCl, pH 7.5, 100 mM NaCl, 10 mM MgCl_2_, and centrifuged in an Sorvall rotor for 100 min at 180,000 g. Fractions (polysomal and subpolysomal) were collected monitoring the absorbance at 254 nm and treated directly with proteinase K. After phenol–chloroform extraction and isopropanol precipitation, polysomal RNA was resuspended in 30 μl of water. RNA quality was assessed by agarose gel electrophoresis and by the Agilent 2100 Bioanalyzer platform.

### Quantitative real-time RT-PCR

Reverse Transcription of RNA to produce cDNA was done on total and polysomal extracts with the Superscript® VILO^TM^ cDNA Synthesis Kit (Invitrogen). TaqMan quantitative real-time PCR was performed in a 10-μL reaction with a KAPA PROBE FAST universal qPCR (Kapa Biosystems). Four genes were used as endogenous controls: ACTB, GADPH, HPRT1, TBP. The geometric mean of the four controls was used to calculate the ΔC_T_ for twelve other genes: MFAP4, TSC22D2, GPM6A, PSAPL1, AG2, EGR1,PCIF1, EGR2, ZNF655, RPL27, SLC2A3, RPL10A . To compare gene expression before and after EGF, the ΔΔC_T_ method was used. All reactions were performed in 3–9 technical replicates for each RNA purified from all the three biological replicates. TaqMan primers and probes used in analyses (purchased from Applied Biosystems) are listed in Additional file 1: Table S1.

### Microarray hybridization and scanning, data acquisition and analysis

Total, polysomal and subpolysomal RNA were hybridized on the Agilent-014850 Whole Human Genome Microarray 4x44K G4112F following the manifacturer’s protocol. Hybridized microarray slides were scanned with an Agilent DNA Microarray Scanner G2505C. μm resolution with the manufacturer’s software (Agilent ScanControl 8.1.3). The scanned TIFF images were analyzed numerically and background corrected using the Agilent Feature Extraction Software version 10.7.7.1 according to the Agilent standard protocol GE1_107_Sep09. The output of Feature Extraction was analyzed with the R software environment for statistical computing (http://htpp://www.r-project.org/) and the Bioconductor library of biostatistical packages (http://www.bioconductor.org/). Low signal Agilent features (11,003), distinguished by a repeated “absent” detection call across the majority of the arrays in every condition, were filtered out from the analysis, leaving 30,075 features corresponding to 15,258 HGNC genes. Signal intensities across arrays were normalized with the quantile normalization algorithm [60]. Signals intensities from probes associated with the same gene were averaged. DEGs were identified with the Rank Product method implemented in the Bioconductor RankProd package (pfp < 0.2 as threshold). All microarray data are available through the Gene Expression Omnibus database (http://www.ncbi.nlm.nih.gov/geo/) using the accession number GSE20277.

### Western blotting

Cells were lysed in Ripa lysis buffer (Tris 50 mM a pH 7.4, NaCl 150 mM, Igepal CA-630 1%, EDTA 1 mM, Na deoxycholate 0.5%) containing protease and phosphatase inhibitors (Sigma-Aldrich). Total cell extracts were diluted in 2X SDS protein gel loading solution, boiled for 5 min, separated on 12% SDS–polyacrylamide gel electrophoresis (SDS–PAGE) and processed following standard procedures. The goat polyclonal antibody anti-phospo-eIF4E (Santa Cruz Biotechnology, Santa Cruz, CA) was diluted at 1:500, the rabbit anti-phospho-Akt (Cell Signaling Technology, Danrers, MA) at 1:1000, the goat anti-beta-actin (Santa Cruz Biotechnology, Santa Cruz, CA) at 1:1000 and the rabbit anti-Myc (Cell Signaling Technology, Danrers, MA) at 1:1000. The nitrocellulose membrane signals were detected by chemiluminescence. Experiments were performed at least three times for each cell preparation.

### Ontological analysis of DEGs

The DAVID resource [61] was used for gene-annotation enrichment analysis of the transcriptome and the translatome DEG lists with categories from the following resources: PIR (http://pir.georgetown.edu/), Gene Ontology (http://www.thegeneontology.org), KEGG (http://www.genome.jp/kegg/) and Biocarta (http://www.biocarta.com/default.aspx) pathway databases, PFAM (http://pfam.sanger.ac.uk/) and COG (http://www.ncbi.nlm.nih.gov/COG/) databases. The significance of overrepresentation was determined at a false discovery rate of 5% with Benjamini multiple testing correction. Matched annotations were used to estimate the uncoupling of functional information as the proportion of annotations overrepresented in the translatome but not in the transcriptome readings and vice versa.

### Data collection, pre-processing and identification of differentially expressed genes (DEGs)

High-throughput data on global changes at the transcriptome and translatome levels were gathered from public data repositories: Gene Expression Omnibus (http://www.ncbi.nlm.nih.gov/geo/), ArrayExpress (http://www.ebi.ac.uk/microarray-as/ae/), Stanford Microarray Database (http://smd.stanford.edu/). Minimum requirements we established for datasets to be included in our analysis were: full access to raw data, hybridization replicas for every experimental condition, two-group comparison (treated group vs. control group) for both transcriptome and translatome. Selected datasets are detailed in Table 1 and Additional file 4. Raw data were treated following the same procedure described in the previous section to determine DEGs in either the transcriptome or the translatome. Additionally, *t*-test and SAM were used as alternative DEGs selection methods applying a Benjamini Hochberg multiple test correction to the resulting p-values.

### Pathway and network analysis with IPA

The IPA software (Ingenuity Systems, http://www.ingenuity.com) was used to assess the involvement of transcriptome and translatome differentially expressed genes in known pathways and networks. IPA uses the Fisher exact test to determine the enrichment of DEGs in canonical pathways. Pathways with a Bonferroni-Hochberg corrected p-value < 0.05 were considered significantly over-represented. IPA also generates gene networks by using experimentally validated direct interactions stored in the Ingenuity Knowledge Base. The networks generated by IPA have a maximum size of 35 genes, and they receive a score indicating the likelihood of the DEGs to be found together in the same network due to chance. IPA networks were generated from transcriptome and translatome DEGs of each dataset. A score of 4, used as a threshold for identifying significant gene networks, indicates that there is only a 1/10000 probability that the presence of DEGs in the same network is due to random chance. Each significant network is associated by IPA to three cellular functions, based on the functional annotation of the genes in the network. For each cellular function, the number of associated transcriptome networks and the number of associated translatome networks across all the datasets was calculated. For each function, a translatome network specificity degree was calculated as the number of associated translatome networks minus the number of associated transcriptome networks, divided by the total number of associated networks. Only cellular functions with more than five associated networks were considered.

### Semantic similarity

To accurately measure the semantic transcriptome-to-translatome similarity, we also adopted a measure of semantic similarity that takes into account the contribution of semantically similar terms besides the identical ones. We chose the graph theoretical approach [62] because it depends only on the structuring rules describing the relationships between the terms in the ontology in order to quantify the semantic value of each term to be compared. Thus, this approach is free from gene annotation biases affecting other similarity measures. Being also specifically interested in distinguishing between the transcriptome specificity and the translatome specificity, we separately computed these two contributions to the proposed semantic similarity measure. In this way the semantic translatome specificity is defined as 1 minus the averaged maximal similarities between each term in the translatome list with any term in the transcriptome list; similarly, the semantic transcriptome specificity is defined as 1 minus the averaged maximal similarities between each term in the transcriptome list and any term in the translatome list. Given a list of *m* translatome terms and a list of *n* transcriptome terms, semantic translatome specificity and semantic transcriptome specificity are therefore defined as:

(1) $$semantic\_translatome\_specificity=1-\frac{\sum_{1\leq i\leq m}\underset{1<=j<=n}{\max(sem.sim._{i,j})}}{m}$$

(2) $$semantic\_transcriptome\_specificity=1-\frac{\sum_{1\leq i\leq n}\underset{1<=j<=m}{\max(sem.sim._{i,j})}}{n}$$

where *sem.sim.* is the semantic similarity between two GO terms. Both transcriptome specificity and translatome specificity range from 0 (no specificity) to 1 (full specificity).

### Calculation of the semantic transcriptome Vs translatome specificity degree associated to GOslim terms

For each GO term the transcriptome specificity degree is calculated as the ratio between the number of datasets in which it is transcriptome specifically over-represented and the number of datasets in which it is over-represented, while the translatome specificity degree is calculated as the ratio between the number of datasets in which it is translatome specifically overrepresented and the number of datasets in which it is overrepresented. According to the GO structure, terms are grouped into the parental GOslim categories and the median transcriptome and translatome specificity degrees are calculated. Within each of the three GO domains, categories were sorted from the most transcriptome specific to the most translatome specific by subtracting the transcriptome specificity degree from the translatome specificity degree.

### Likelihood ratio test

The Likelihood Ratio test was used to test the null hypothesis that DEG numbers are the same between transcriptome and translatome, against the alternative hypothesis that they can be different.

### Random overlap test

For each dataset, n1 and n2 genes were randomly extracted from the population of DEGs (n1 and n2 being the real numbers of observed transcriptome and translatome DEGs for the dataset). The number of common genes was calculated as the random overlap and the extraction process was repeated 1 million times. The overlap test calculates the probability of the observed overlap to be higher than the random overlap.

### Mutual information test

Mutual information is used in each dataset to measure the mutual dependence between being a transcriptome DEGs and being a translatome DEG. Each of the two variables is discrete, taking the value of 1 if the gene is differentially expressed, 0 if the gene is not differentially expressed. Minimal mutual information for each dataset is calculated as the case in which the two lists of n1 transcriptome DEGs and n2 translatome DEGs have null overlap. Maximal mutual information is calculated as the case in which the two lists of DEGs are completely overlapping and have size (n1 + n2)/2. Random mutual information is calculated for each dataset from one million of random extractions, similarly as described in the previous section. The mutual information test calculates the probability of the observed mutual information to be higher than the random mutual information.

## Abbreviations

DEGs, differentially expressed genes; GO, Gene Ontology; MHT, multiple hypotheses testing; miRNA, microRNA; ncRNA, noncoding RNA; RBP, RNA binding protein; UTR, untranslated region; TOP, terminal oligo-pyrimidine.

## Misc

Toma Tebaldi and Angela Re equal contributors.

## Competing interests

The authors declare that they have no competing interests.

## Authors’ contributions

TT and AR collected and analyzed the data. GV and IP performed the EGF experiments. AP and EB suggested and supervised the statistical analysis. AQ supervised the whole project. AQ, TT and AR wrote the paper. TT realized the graphics. All authors discussed the results and implications and commented on the manuscript at all stages. All authors read and approve the final manuscript.

## Supplementary Material

Additional file 1 Contains Supplementary Figures S1:S5 and Supplementary Tables S1:S3. Click here for file

Additional file 2 Contains the complete microarray data for the EGF experiment, with the results of the DEGs analysis for both the transcriptome and the translatome. Click here for file

Additional file 3 Contains the complete ontological enrichment analysis of the EGF experiment for both the transcriptome and the translatome. Click here for file

Additional file 4 Contains the detailed description of all the reanalyzed datasets included in our survey. Click here for file

Additional file 5 Contains the complete microarray data of all the reanalyzed datasets included in our survey, with the results of the DEGs analysis for both the transcriptome and the translatome. Click here for file

Additional file 6 Contains the complete ontological enrichment analysis of all the reanalyzed datasets included in our survey, for both the transcriptome and the translatome. Click here for file

Additional file 7 Contains the complete IPA pathway enrichment analysis of all the reanalyzed datasets included in our survey, for both the transcriptome and the translatome. Click here for file

Additional file 8 Contains the complete IPA network analysis of all the reanalyzed datasets included in our survey, for both the transcriptome and the translatome. Click here for file
