# Supplementary material for: Widespread uncoupling between transcriptome and translatome variations after a stimulus in mammalian cells
Source: BMC Genomics. 2012 Jun 6;13:220. doi: 10.1186/1471-2164-13-220 (PMC3441405; doi:10.1186/1471-2164-13-220)
Supplement: Additional file 1 — Contains Supplementary Figures S1:S5 and Supplementary Tables S1:S3. [file 1471-2164-13-220-S1.doc]

**
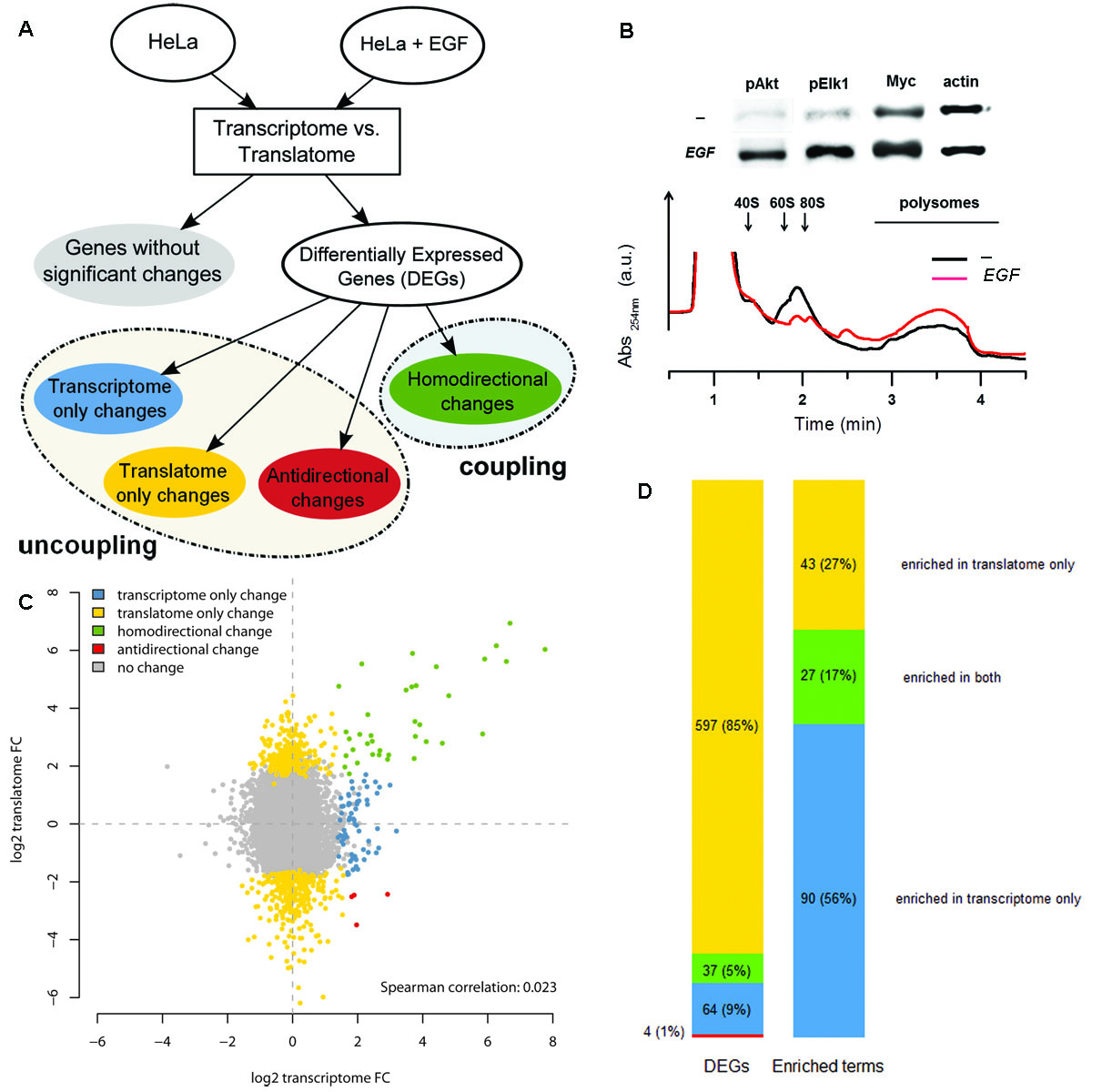
**

**Supplementary Figure S1.** Numerical uncoupling between transcriptome and translatome associated functional over represented terms. Terms enriched only in transcriptome DEGs are displayed in cyan, terms enriched only in translatome DEGs are displayed in yellow, terms enriched in both transcriptome and translatome DEGs are displayed in green.

**
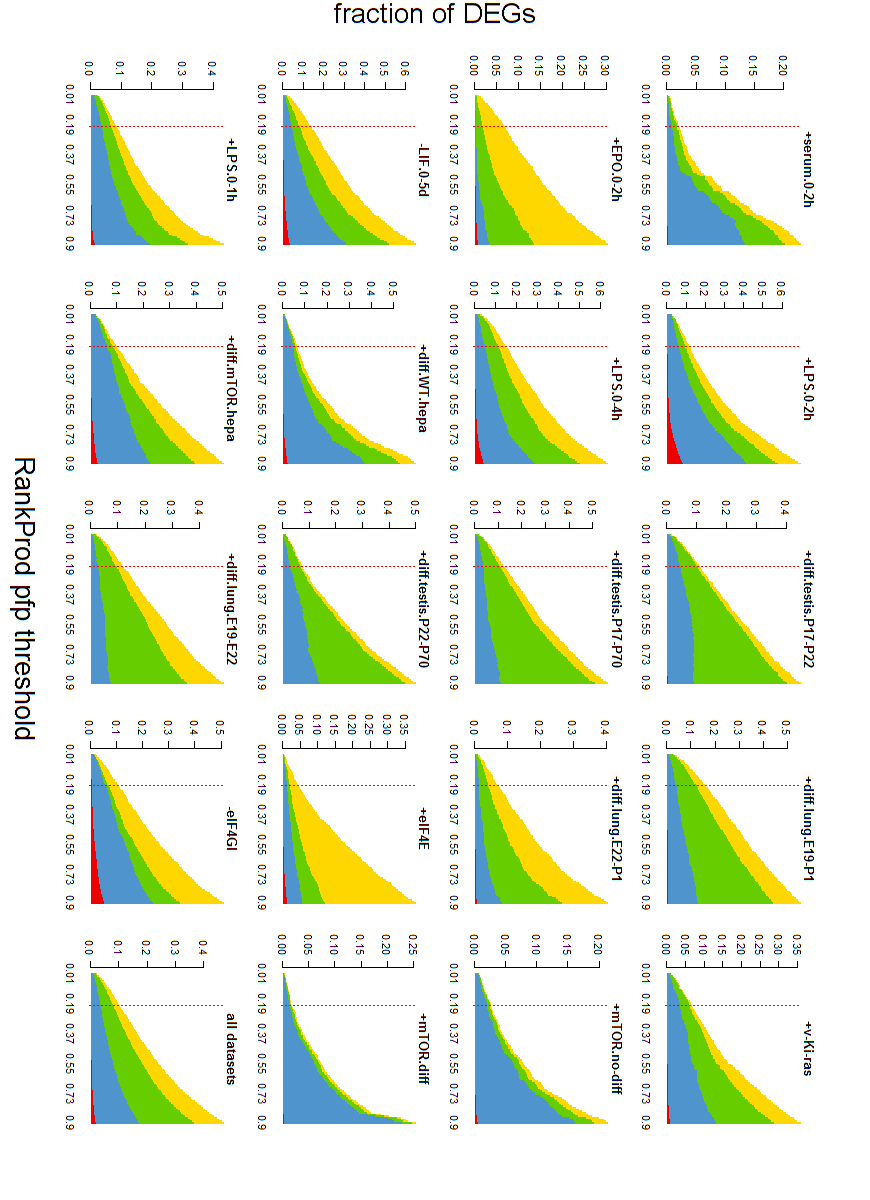
**

**Supplementary Figure S2.** Fraction of genes detected as DEGs (y axis) at different RankProd pfp threshlolds (x axis). One graphic is displayed for each dataset. The last graphic shows the behavior of all the datasets together. Uncoupling and coupling classes of DEGs are color labeled: genes with significant variations only in the transcriptome (in cyan), genes with significant variations only in the translatome (in yellow), genes with opposite significant variations between transcriptome and translatome (in red), genes with homodirectional significant variations (in green).


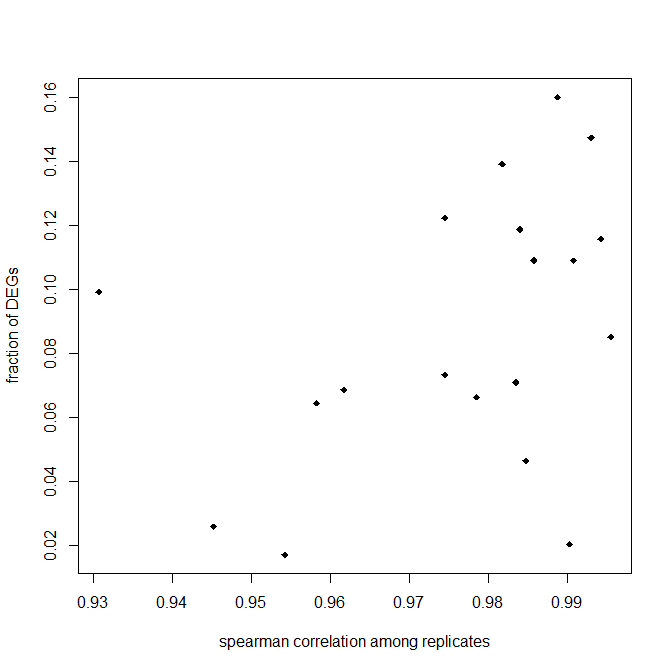


**Supplementary Figure S3.** Independence between the fraction of detected DEGs and the average Spearman correlation among replicas. For each transcriptome - translatome dataset of the collection, the average correlation among replicas is plotted on the x axis, while the fraction of genes detected as DEGs is plotted on the y axis.

**
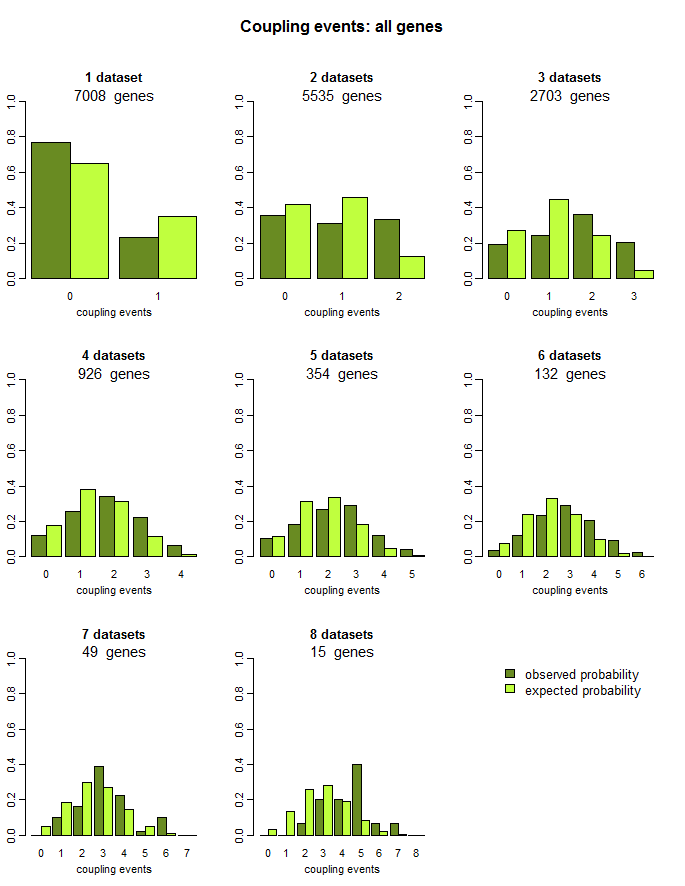
**

**Supplementary Figure S4.** Observed coupling frequencies are higher than what expected by chance. All human, rat and mouse genes were divided in eight classes according to the number n1 of their occurrences as DEGs across all the collected datasets (n1 ranging from a minimum of 1 to a maximum of 8). For each gene, the number of occurrences as coupled was next counted. The resulting distribution probabilities of coupling events are displayed in dark green. Expected probabilities were calculated as multinomial distributions with n1 numbers of trials and coupling probability = 0.35 (the average percentage of coupled DEGs observed across all the datasets). The observed distributions are always higher than the expected distributions, suggesting the presence of gene specific mechanisms that couple transcriptional and translational variations in a broad set of conditions.

**
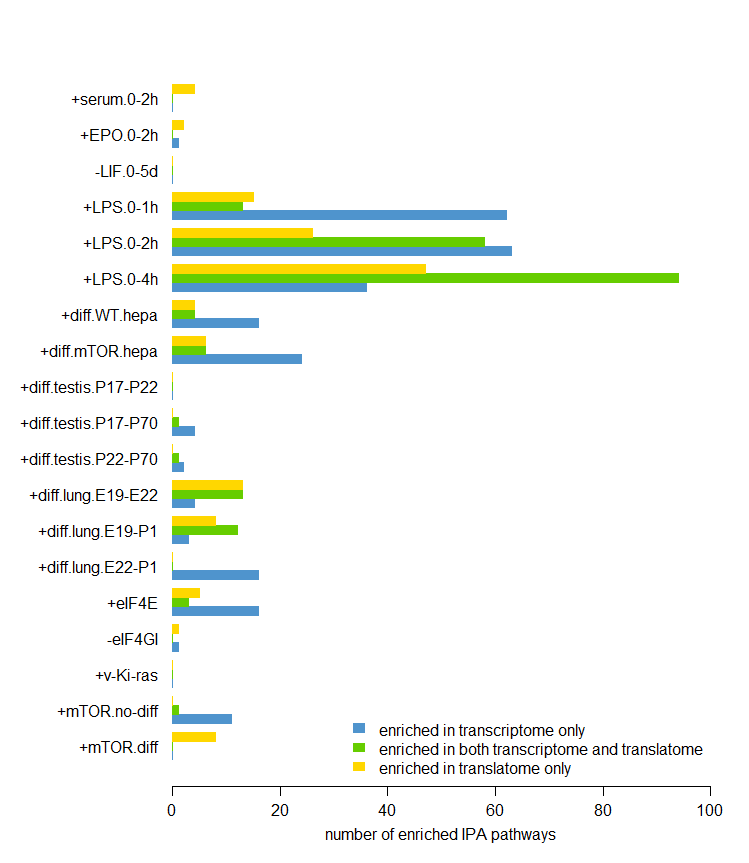
**

**Supplementary Figure S5.** Uncoupling between significantly enriched IPA canonical pathways. For each dataset, the number of pathways significantly enriched only for transcriptome DEGs (in cyan), the number of pathways significantly enriched only for translatome DEGs (in yellow) and the number of pathways enriched for both transcriptome and translatome DEGs (in green) are displayed. Three datasets have no significantly enriched pathways. In 14 out of the 16 remaining datasets, the number of specific pathways exceeds the number of common pathways.

| **Gene Symbol** | **Assay ID** | **Transcriptome log2 FC** | **Translatome log2 FC** |  |  |
| --- | --- | --- | --- | --- | --- |
| GPM6A | Hs01009142_m1 | 0.70 | 1.28 |  |  |
| PSAPL1 | Hs00999475_s1 | 0.10 | 2.06 |  |  |
| AGR2 | Hs00180702_m1 | 0.14 | -0.08 |  |  |
| EGR1 | Hs00152928_m1 | 3.94 | 7.93 |  |  |
| PCIF1 | Hs00363733_m1 | 0.01 | -0.22 |  |  |
| EGR2 | Hs00166165_m1 | 3.30 | 6.98 |  |  |
| ZNF655 | Hs01002760_m1 | 0.23 | 0.09 |  |  |
| RPL27 | Hs03044962_g1 | -0.18 | 0.13 |  |  |
| SLC2A3 | Hs00359840_m1 | 0.64 | 0.77 |  |  |
| RPL10A | Hs03043870_g1 | 0.03 | -0.32 |  |  |
| ACTB | Hs99999903_m1 | -0.17 | 0.38 |  |  |
| GAPDH | Hs02758991_g1 | -0.02 | -0.17 |  |  |
| HPRT1 | Hs01003267_m1 | 0.06 | -0.11 |  |  |
| TBP | Hs00427620_m1 | 0.11 | -0.15 |  |  |

**Supplementary Table S1.** TaqMan® Gene Expression Assays (Applied Biosystems) and results.

| **Affymetrix Platform ID** | **# probesets on chip** | **# unambiguous probesets** | **% of mapping loss** | **# ENSEMBL genes** | **# avg probesets for gene** |
| --- | --- | --- | --- | --- | --- |
| ***Human*** |  |  |  |  |  |
| **HG-U133_Plus_2** | 54675 | 36666 | 33% | 16440 | 2.23 |
| **HG-U133A_2** | 22277 | 19726 | 11% | 11570 | 1.70 |
| **HG_U95Av2** | 12625 | 11399 | 10% | 8100 | 1.41 |
| **HG-U133A** | 22283 | 19730 | 11% | 11550 | 1.71 |
| ***Mouse*** |  |  |  |  |  |
| **MG_U74Av2** | 12488 | 11174 | 11% | 8250 | 1.35 |
| **Mouse430_2** | 45101 | 33900 | 25% | 16800 | 2.02 |
| **MOE430A** | 22690 | 21186 | 7% | 12390 | 1.71 |
| ***Rat*** |  |  |  |  |  |
| **Rat230_2** | 31099 | 18314 | 41% | 12010 | 1.52 |

**Supplementary Table S2.** Differences among Affymetrix chips during the preprocessing stage of data analysis. The table shows the number of probesets on the chip, the number of unambiguous probesets, the percentage of probesets lost after the mapping step, the number of mapped ENSEMBL genes and the average number of probesets mapping to a gene.

| **Cellular function** | **Number of transcriptome networks** | **Number of translatome networks** | **Translatome**  **network**  **specificity** |
| --- | --- | --- | --- |
| *Metabolic Disease* | 1 | 6 | 0.71 |
| *Neurological Disease* | 3 | 11 | 0.57 |
| *Cellular Compromise* | 2 | 6 | 0.50 |
| *Free Radical Scavenging* | 2 | 6 | 0.50 |
| *RNA Post-Transcriptional Modification* | 4 | 9 | 0.38 |
| *Developmental Disorder* | 5 | 11 | 0.38 |
| *Skeletal and Muscular Disorders* | 2 | 4 | 0.33 |
| *Genetic Disorder* | 17 | 25 | 0.19 |
| *Gene Expression* | 37 | 53 | 0.18 |
| *Endocrine System Development and Function* | 5 | 7 | 0.17 |
| *Drug Metabolism* | 9 | 12 | 0.14 |
| *Energy Production* | 6 | 8 | 0.14 |
| *Cell Death* | 52 | 66 | 0.12 |
| *Cardiovascular Disease* | 4 | 5 | 0.11 |
| *Glutathione Depletion In Liver* | 5 | 6 | 0.09 |
| *Cell-To-Cell Signaling and Interaction* | 41 | 48 | 0.08 |
| *Cell Morphology* | 12 | 14 | 0.08 |
| *Tissue Development* | 26 | 30 | 0.07 |
| *Cellular Development* | 39 | 43 | 0.05 |
| *Cellular Growth and Proliferation* | 37 | 40 | 0.04 |
| *Cellular Assembly and Organization* | 52 | 56 | 0.04 |
| *Cell Cycle* | 49 | 49 | 0.00 |
| *Cell Signaling* | 15 | 15 | 0.00 |
| *Connective Tissue Development and Function* | 13 | 13 | 0.00 |
| *Antimicrobial Response* | 7 | 7 | 0.00 |
| *Organismal Injury and Abnormalities* | 7 | 7 | 0.00 |
| *Nervous System Development and Function* | 6 | 6 | 0.00 |
| *Organ Development* | 5 | 5 | 0.00 |
| *Connective Tissue Disorders* | 4 | 4 | 0.00 |
| *Reproductive System Development and Function* | 4 | 4 | 0.00 |
| *RNA Trafficking* | 3 | 3 | 0.00 |
| *Hematological System Development and Function* | 36 | 35 | -0.01 |
| *Inflammatory Response* | 28 | 26 | -0.04 |
| *Infectious Disease* | 12 | 11 | -0.04 |
| *Lipid Metabolism* | 48 | 43 | -0.05 |
| *Protein Synthesis* | 16 | 14 | -0.07 |
| *Skeletal and Muscular System Development and Function* | 8 | 7 | -0.07 |
| *Nucleic Acid Metabolism* | 8 | 7 | -0.07 |
| *Amino Acid Metabolism* | 7 | 6 | -0.08 |
| *Cancer* | 40 | 34 | -0.08 |
| *Cellular Movement* | 48 | 40 | -0.09 |
| *Immune Cell Trafficking* | 12 | 10 | -0.09 |
| *Endocrine System Disorders* | 6 | 5 | -0.09 |
| *Protein Degradation* | 6 | 5 | -0.09 |
| *Embryonic Development* | 22 | 18 | -0.10 |
| *Protein Folding* | 5 | 4 | -0.11 |
| *Small Molecule Biochemistry* | 65 | 50 | -0.13 |
| *DNA Replication* | 42 | 32 | -0.14 |
| *Immunological Disease* | 4 | 3 | -0.14 |
| *Molecular Transport* | 45 | 33 | -0.15 |
| *Cellular Function and Maintenance* | 40 | 28 | -0.18 |
| *Tissue Morphology* | 10 | 7 | -0.18 |
| *Carbohydrate Metabolism* | 16 | 11 | -0.19 |
| *and Repair* | 16 | 11 | -0.19 |
| *Reproductive System Disease* | 6 | 4 | -0.20 |
| *Recombination* | 30 | 19 | -0.22 |
| *Post-Translational Modification* | 21 | 13 | -0.24 |
| *Gastrointestinal Disease* | 13 | 8 | -0.24 |
| *Hematopoiesis* | 5 | 3 | -0.25 |
| *Hematological Disease* | 11 | 6 | -0.29 |
| *Inflammatory Disease* | 6 | 3 | -0.33 |
| *Dermatological Diseases and Conditions* | 9 | 4 | -0.38 |
| *Cardiovascular System Development and Function* | 14 | 6 | -0.40 |
| *Antigen Presentation* | 7 | 3 | -0.40 |
| *Vitamin and Mineral Metabolism* | 7 | 2 | -0.56 |
| *Organismal Development* | 13 | 3 | -0.63 |

**Supplementary Table S3**. Cellular functions associated to IPA networks across all the datasets. Each significant network built by IPA from transcriptome or translatome DEGs is associated to three cellular functions, based on the functional annotation of the nodes of the network. For each cellular function, the number of associated transcriptome networks and the number of associated translatome networks across all the datasets was calculated. For each function, a translatome network specificity degree was calculated as the number of associated translatome networks minus the number of associated transcriptome networks, divided by the total number of associated networks. Only cellular functions with more than five associated networks were considered. In the table, cellular functions are ordered from the most translatome specific to the most transcriptome specific.
